# Small cells – big issues: biological implications and preclinical advancements in small cell lung cancer

**DOI:** 10.1186/s12943-024-01953-9

**Published:** 2024-02-24

**Authors:** Anna Solta, Büsra Ernhofer, Kristiina Boettiger, Zsolt Megyesfalvi, Simon Heeke, Mir Alireza Hoda, Christian Lang, Clemens Aigner, Fred R. Hirsch, Karin Schelch, Balazs Döme

**Affiliations:** 1 Department of Thoracic Surgery, Comprehensive Cancer Center, Medical University of Vienna, Waehringer Guertel 18-20, 1090 Vienna, Austria; 2 Department of Thoracic Surgery, Semmelweis University and National Institute of Oncology, Budapest, Hungary; 3 National Koranyi Institute of Pulmonology, Budapest, Hungary; 4 Department of Thoracic Head and Neck Medical Oncology, The University of Texas MD Anderson Cancer Center, Houston, TX USA; 5 Division of Pulmonology, Department of Medicine II, Medical University of Vienna, Vienna, Austria; 6 Division of Medical Oncology, University of Colorado Anschutz Medical Campus, Aurora, CO USA; 7 Center for Thoracic Oncology, Mount Sinai Health System, Tisch Cancer Institute, New York, NY USA; 8 Center for Cancer Research, Medical University of Vienna, Vienna, Austria; 9 Department of Translational Medicine, Lund University, Lund, Sweden

**Keywords:** Small cell lung cancer, Molecular subtypes, Translational progress, Preclinical models

## Abstract

Current treatment guidelines refer to small cell lung cancer (SCLC), one of the deadliest human malignancies, as a homogeneous disease. Accordingly, SCLC therapy comprises chemoradiation with or without immunotherapy. Meanwhile, recent studies have made significant advances in subclassifying SCLC based on the elevated expression of the transcription factors ASCL1, NEUROD1, and POU2F3, as well as on certain inflammatory characteristics. The role of the transcription regulator YAP1 in defining a unique SCLC subset remains to be established. Although preclinical analyses have described numerous subtype-specific characteristics and vulnerabilities, the so far non-existing clinical subtype distinction may be a contributor to negative clinical trial outcomes. This comprehensive review aims to provide a framework for the development of novel personalized therapeutic approaches by compiling the most recent discoveries achieved by preclinical SCLC research. We highlight the challenges faced due to limited access to patient material as well as the advances accomplished by implementing state-of-the-art models and methodologies.

## Introduction

Small cell lung cancer (SCLC) is the most aggressive histologic subtype of lung cancer and accounts for roughly 13–15% of all lung cancers [1]. The recalcitrant nature of SCLC is demonstrated by rapid disease progression accompanied with early metastatic manifestation [2]. More than two-thirds of patients already present with advanced disease at the time of diagnosis [3]. SCLC is initially highly sensitive to doublet cytotoxic chemotherapy with or without radiotherapy, resulting in response rates of more than 60%. However, disease recurrence and acquired resistance occur in almost every patient. In the recent past, immune checkpoint blockade has extended the therapeutic regimen of SCLC. However, the survival of SCLC patients remains poor with a 5-year survival rate of below 7% [2]. Although current preclinical evidence suggests the existence of four distinct molecular subtypes of SCLC, the clinical guidelines still classify SCLC as a single disease entity [4]. The most recent clinical advancements in SCLC have been extensively elaborated by Megyesfalvi et al. [5]. This comprehensive review aims to summarize the biological milestones in characterizing SCLC that have been achieved by preclinical research. Novel therapeutic approaches based on patient stratification are an unmet need to improve the clinical success of SCLC. Emphasis is laid on past accomplishments and new avenues in order to aid in improving patient outcomes.

### Advancements achieved by preclinical research

#### Cell of origin

The lining of the lung epithelia is compartmentalized along the proximal-to-distal axis into three structurally and functionally distinct domains [6]. The transition between the airways and the alveoli, (bronchioalveolar duct junction, BADJ) contains bronchioalveolar stem cells (BASCs) that give rise to airway and alveolar cells [7]. The most distal alveolar region consists of squamous alveolar type 1 (AT1) cells mediating gas exchange and cuboidal alveolar type 2 (AT2) cells that secrete surfactant to prevent alveolar collapse [8]. AT2 cells function as stem cells that contribute to alveolar renewal and repair due to their self-perpetuating ability and the transition into AT1 cells [9]. Basal cells are progenitor cells of ciliated, neuroendocrine (NE), and club cells found in the proximal airways [10]. Clusters of NE cells represent extremely rare cell populations characterized by the expression of the basic-helix-loop-helix (bHLH) transcription factor (TF) Inhibitor of Differentiation 2 (ID2) [11]. ID2 regulates mitochondrial activities; its upregulation assists SCLC cells in gaining sufficient energy to support fast mitosis and proliferation [12]. Accordingly, NE cells emerge in lung organogenesis and are enriched in fetal and neonatal lungs [13]. In adult intrapulmonary airways, approximately 0.41% of pulmonary neuroendocrine cells (PNECs) are present [14]. PNECs are commonly arranged in small cell clusters known as neuroepithelial bodies (NEBs) [15, 16]. PNECs share characteristics of neuronal and endocrine cells, such as comprising the synthesis, accumulation, and release of serotonin, gastrin-releasing peptide (GRP), neuron-specific enolase (NSE) and other transmitters such as bombesin [16]. Interestingly, calcitonin gene-related peptide (CGRP), neural cell adhesion molecule 1 (NCAM1), and mammalian achaete-scute complex homolog 1 (MASH1/ASCL1) not only play important roles in neuronal differentiation, but are also highly expressed in PNECs [15].

**Fig. 1:**
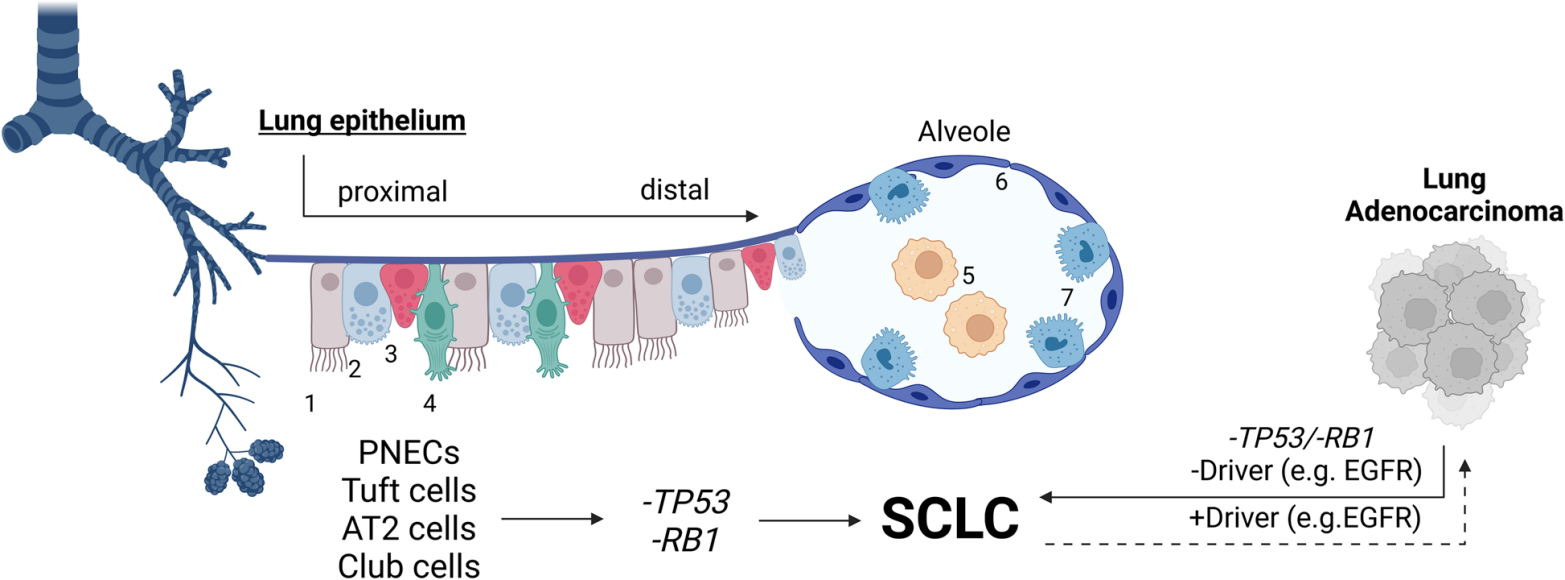
SCLC tumorigenesis from different cells of origin

Based on recent evidence, SCLC may arise most frequently from PNECs, but could also emerge from lung epithelial cells such as basal or club cells and AT2 cells in certain cases (Fig. 1). Recent findings suggest that tuft cells also act as putative progenitor cells in a specific subtype of SCLC [17]. Tuft cells (also termed brush cells) were discovered in rodents more than half a decade ago and were defined as chemosensory cells of the lung epithelial lining [18]. Notably, tuft cells mediate chemosensory, immune, and neuronal pathways. However, the latter two are not commonly linked to epithelial cell function [19]. Loss-of-function mutations including tumor protein 53 (*TP53*) and retinoblastoma 1 (*RB1*) in PNECs, tuft, club, or AT2 cells evidently evoke SCLC development. Moreover, SCLC may also transdifferentiate from lung adenocarcinoma (LUAD) following loss-of-driver mutations such as epidermal growth factor receptor (EGFR) (Fig. 1).

Normal lung epithelium consists of a variety of cells including ciliated cells (1), club cells (2), PNECs (3), tuft cells (4), alveolar macrophages (5), AT1 pneumocytes (6), and AT2 pneumocytes (7). Upon *TP53* and *RB1* loss, several cell types of the lung epithelium may transform into SCLC. Of note, lung epithelial cells may also result in lung adenocarcinoma due to driver mutations, which may ultimately transform into SCLC. Created with BioRender.com. AT1/2 - Alveolar type 1/2 cells, EGFR - Epidermal growth factor receptor, PNEC - Pulmonary neuroendocrine cells, RB1 - Retinoblastoma 1, TP53 - Tumor protein 53

#### Genomic alterations

According to genome-wide studies, it is known that tobacco smoking induces the development of almost all lung cancer types [20, 21]. Current or former excessive smoking is related to increased tumor mutational burden (TMB), heterogeneity, and driver mutations [22]. Acquired chromosomal abnormalities were identified in several SCLC specimens [23]. Allele loss involving the chromosome arm 3p is almost universally detected in early lung cancer pathogenesis, and may, therefore, affect several potential tumor suppressor (TS) genes. SCLC is characterized by almost universal, bi-allelic inactivation of both TS genes *TP53* and *RB1* [24]. Therefore, the loss of *TP53* (75%-90% of patients [25]) and *RB1* (nearly 100% [26]) is confirmed in the vast majority of cases. However, therapeutic targeting is still not feasible [27]. Genetically engineered SCLC mouse models (GEMMs) initiated by loss of *TP53* and *RB1* present multiple aggressive lung tumors with striking similarities to human disease. Further alterations involving loss of TF phosphatase and tensin homolog (*PTEN*) or nuclear factor 1B (*NFIB*) add to the aggressive behavior of SCLC [28].

Additional alterations of oncogenic drivers of SCLC comprise members of the MYC proto-oncogenes, including three distinct TFs: C-MYC, L-MYC, and N-MYC [24]. All MYC members belong to the superfamily of bHLH leucine zipper TFs that bind to the canonical E-box DNA element [29]. All are paralogs with structural homology and their functional differences are associated with phenotypic disparities [30]. Transcriptional activation of MYC depends on heterodimeric complex formation with MAX proteins and recruitment of TFs. Other mechanisms of activation are arbitrated by histone acetyltransferases (HAT), chromatin remodeling enzymes, and RNA polymerases [31]. C-MYC, the most frequently dysregulated family member, is mediating the cell cycle and transcriptional response, hence, promoting cell growth and proliferation [32].

The TF NFIB is part of the NFI gene complex essential for embryonic development of the lung, kidney, and brain [33]. NFIB is commonly amplified in SCLC and functions as an oncogene driving initiation, progression, and metastasis [34, 35]. SCLC metastases portray changes in profound chromatin accessibility and differences in gene expression programs in comparison to the primary tumor [36]. Accordingly, a pro-metastatic switch promotes neuronal gene expressions related to migration, adhesion, and neuronal differentiation [36]. Hence, high NFIB levels lead to expansion of a poorly differentiated E-cadherin (CDH1)-negative invasive tumor subpopulation, which consequently correlates with worse patient survival [34].

#### Neuroendocrine features of SCLC

Two SCLC phenotypes based on the morphology and growth behavior were described more than 30 years ago [37, 38]. High-NE tumors showed growth characteristics of irregular floating clusters, although some appeared as adherent subpopulations in vitro. By contrast, tumors of low-NE differentiation displayed loose attachment properties or semi-attached, loosely aggregated or single floating cell aggregates (“Indian file” pattern) [39]. Importantly, NE-high cell lines show a “classic” phenotype characterized by small cells with blurred cell borders, fine granular “salt and pepper” chromatin, and small nucleoli [24]. In comparison, the NE-low “variant” group is associated with slightly larger cells displaying distinct cell borders and prominent nucleoli. They exhibit partial or complete loss of NE cell features but show large numbers of inflammatory cells [37, 38]. The intertumoral heterogeneity of NE expression patterns is characterized by changes in morphology, growth properties, and genetic alterations as well as immune and inflammatory responses [40]. While NE-high SCLCs demonstrate a low infiltrating cellular immune response and display an immune desert phenotype, NE-low SCLCs are associated with increased immune cell infiltration and are referred to as immune oasis [41]. Accordingly, non-tuft cell-derived NE-low SCLC patients show better response to immunotherapy compared to NE-high SCLC patients who exhibit low numbers of immune cells and decreased or absent expression of programmed death-ligand 1 (PD-L1) [1, 39, 40].

#### SCLC molecular subtypes are defined by transcription factor expression

**Fig. 2:**
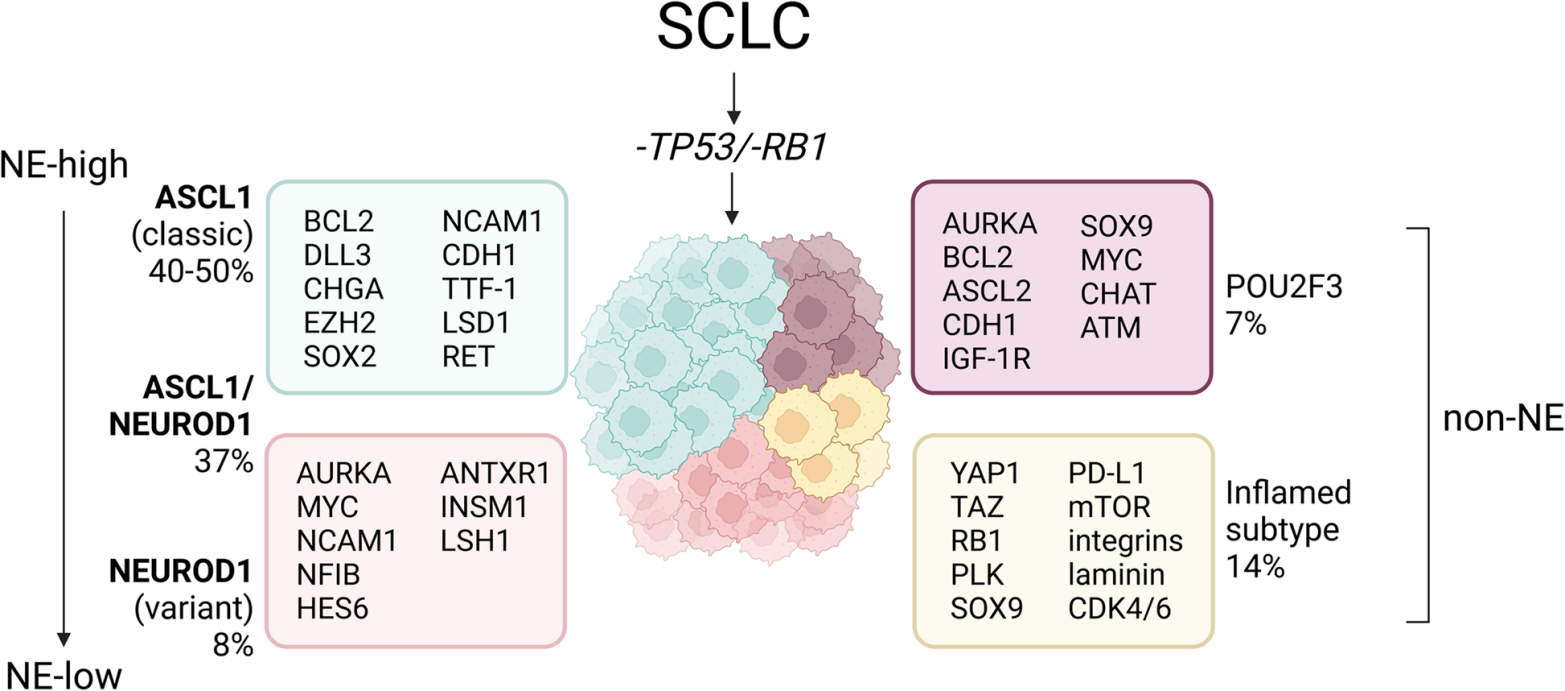
Molecular subclassification of SCLC.  SCLC subclassification discriminates between NE subtypes which are represented by ASCL1- or NEUROD1-driven tumors and non-NE subtypes characterized by POU2F3 or inflamed expression patterns. Distinct expression profiles show potential vulnerabilities of each SCLC subtype. Created with BioRender.com. TP53—Tumor protein 53; RB1 - Retinoblastoma 1; NE - neuroendocrine; PNEC - Pulmonary NE cells; ASCL1 - Achaete-scute homologue 1; NEUROD1 - Neurogenic differentiation factor 1; POU2F3 - POU class 2 homeobox 3; YAP1 - Yes-associated protein 1, QN - Quadruple negative, BCL2 - B-cell lymphoma 2, DLL3 - Delta-like protein 3, CHGA – Chromogranin A, EZH2 - Enhancer of zeste homologue 2, SOX2 - SRY-box transcription factor 2, CDH1 – E-cadherin, TTF-1 - Homeobox protein Nkx2.1, LSD1 - Lysine demethylase 1A, RET - Ret proto-oncogene, AURKA – Aurora kinase A, MYC – MYC proto-oncogene, NCAM1 - Neural cell adhesion molecule 1, NFIB - Nuclear factor 1 B, HES6 - Hairy and enhancer of split 6, ANTXR1 - Anthrax toxin receptor 1, INSM1 - Insulinoma-associated protein 1, ASCL2 - Achaete-scute homologue 2, IGF-1R - Insulin-growth factor receptor 1, SOX9 - SRY-box transcription factor 9, CHAT - Choline O-acetyltransferase, ATM - ATM serine/threonine kinase, TAZ - Homologue to YAP1, PLK - Polo-like kinase, PD-L1 - Programmed death-ligand 1, mTOR – Rapamycin, CDK4/6 - Cyclin-dependent kinases 4/6

Recently, a worldwide resurgence of genomic profiling studies in SCLC including comprehensive molecular analyses of representative preclinical models including cell lines, patient-derived xenografts (PDX), and GEMMs arose [24]. Recent work proposed SCLC molecular subtypes (SCLC-A, SCLC-N, SCLC-P, and SCLC-Y) based on the differential expression of the key transcriptional regulators ASCL1, NEUROD1 (neurogenic differentiation factor 1), POU2F3 (POU class 2 homeobox 3), and YAP1 (yes-associated protein 1) (Fig. 2). Of note, the most recent nomenclature also directs towards an “inflamed” SCLC (SCLC-I) [4, 5]. The association between these subtype-specific TFs includes distinct degrees of NE differentiation. The correlation between individual characteristics and therapeutic vulnerabilities may be of clinical importance [2].

#### Neuroendocrine subtypes (ASCL1 and NEUROD1)

ASCL1-high tumors express NE markers and show classic morphology, whereas NEUROD1-high tumors have lower NE expression and variant morphology [24, 37]. ASCL1 and NEUROD1, two bHLH TFs with pivotal roles in neuronal function and maturation of PNECs, are lineage-specific oncogenes in SCLC. Both TFs act as master regulators of NE differentiation [42, 43]. ASCL1 regulates oncogenic genes including *L-MYC*, *RET*, SRY-box transcription factor 2 (*SOX2)*, *NFIB*, B-cell lymphoma 2 (*BCL2),* and the lung development genes *FOXA2* and *TTF-1*. The homeobox protein Nkx2.1 (NKX2-1/TTF1) is a TF located in club and AT2 cells. TTF1 is fundamental for the development of NE cells and shows higher expression in SCLC-A than in SCLC-N [44]. NE markers such as insulinoma-associated protein 1 (*INSM1)*, *NCAM1*, chromogranin A (*CHGA)*, synaptophysin (*SYP),* and *CGRP* are further direct transcriptional targets of ASCL1 [42]. Additionally, ASCL1 directs members of the Notch pathway including delta like canonical notch ligand 3 (DLL3), while NEUROD1 transcriptional targets include the receptor tyrosine kinase insulin-growth factor receptor 1 (IGF1R) [42, 45]. SCLC-A is highly associated with L-MYC, whereas the SCLC-N subtype is related to the upregulation of C-MYC [4, 46]. Both molecular subtypes are therapeutically relevant as MYC-driven SCLC is particularly sensitive to aurora kinase (AURK) A/B inhibition and C-MYC inhibition may enhance the therapeutic efficacy of chemotherapy [4, 47]. Furthermore, NEUROD1 effectors promote survival, migration, and proliferation of SCLC cells through cell surface receptor tyrosine kinase tropomyosin-related kinase B (TRKB) and NCAM1. Interestingly, TRKB and NCAM1 promote metastasis by inducing invasive behavior in NE lung cancers [48, 49]. Enforced overexpression of TRKB results in altered expression of molecular mediators of epithelial-to-mesenchymal transition (EMT) including downregulation of CDH1 and upregulation of Twist [50]. NCAM1 expression stimulates cell–matrix and neurite outgrowth by regulating fibroblast growth factor (FGF)-receptor (FGFR) interaction. Furthermore, amplification of FGFR1 is found in 5.6% of SCLCs which represents a therapeutic target of interest [1, 51].

ASCL1-driven SCLCs can further be subdivided based on hairy and enhancer of split-1 (HES1) expression. Accordingly, ASCL1-high tumors can be subclustered into HES1-low and HES1-high groups (SCLC-A and SCLC-A2, respectively) [3, 4, 52]. HES1 is a downstream target of the Notch pathway and acts as an important regulator of cell proliferation, differentiation, invasion, cancer stem cell (CSC)-like properties, and tumorigenicity [53, 54]. Of note, drug screening indicated that SCLC-A2 (described as NEv2) is more resistant to AURK and mammalian target of rapamycin (mTOR) inhibition [52].

The NE subtypes share the expression of the TF INSM1, a driver of NE differentiation. SCLC-A and -N are both associated with chemosensitivity in SCLC cell lines [4, 45]. However, SCLC-A is suspected to be even more chemosensitive than SCLC-N [37]. Clinical data demonstrated that ASCL1 overexpression is a negative prognostic indicator in early-stage SCLC patients and is associated with poor prognosis in surgically-resected SCLCs [55]. A subset of SCLCs lacks expression of either ASCL1 or NEUROD1, and some of these double-negative tumors are highly dependent on lower or absent expression of INSM1 [24, 42]. In combination with the lack of NE markers, these tumors are classified as the non-NE SCLC subtypes P and I [4, 56].

#### Non-neuroendocrine subtypes (POU2F3 and “inflamed” subtype)

The class II POU domain transcription factor POU2F3 (also known as OCT11 and SKN-1a) has been reported to be a master regulator of normal and malignant tuft cell fate [17, 57, 58]. These chemosensory cells are found in the epithelial lining of the gastrointestinal and respiratory tracts [59, 60]. POU2F3-high tumors lack typical NE markers, their divergent expression patterns resemble tuft cell signatures, suggesting that SCLC-P arises from a distinct cell of origin [17]. SCLC-P tumors occasionally show low levels of NE markers such as GRP and calcitonin related polypeptide alpha (CALCA; encoding CGRP1) and lineage-specific TFs of tuft cells comprising SOX9 and ASCL2 [4, 17, 61]. Other prominent markers in SCLC-P include IGF-1R and growth factor independence 1B (GFI1B) [45, 62]. GFI and its homolog GFI1B modulate transcriptional repression through the binding of its cofactor lysine demethylase 1A (LSD1/KDM1A). However, the therapeutic benefit of LSD1 inhibition is associated with Notch activation and suppression of ASCL1 in SCLC. This indicates a potential vulnerability in SCLC-A [63]. SCLC-P is associated with improved survival outcomes compared to the other subtypes, however, further personalized approaches are needed [17, 24, 41].

The inflamed SCLC subtype (SCLC-I) exhibits a mesenchymal and inflammatory phenotype with increased expression of human leukocyte antigens (HLAs), interferon-γ (IFN-γ) activation, and immune checkpoints, consistent with the association between EMT- and immune-related gene expressions [64]. Furthermore, SCLC-I expresses low levels of the epithelial marker CDH1 and high levels of the mesenchymal markers vimentin and AXL [64]. SCLC-I tumors also highly express immune checkpoint molecules including PD-L1, PD-1, and cytotoxic T-lymphocyte-associated protein 4 (CTLA4). SCLC-I experiences the greatest benefit from immuno-chemotherapy [64]. Beforehand, the fourth subtype was suspected to be YAP1-driven, however, its existence is currently being disputed and its clinical context remains unclear [65], since YAP1 can only be confidently detected in vitro cell culture settings. Of note, recent immunohistochemistry (IHC)-based studies provided evidence for a quadruple-negative SCLC subtype (SCLC-QN), which is characterized by low/absent expression of all four TFs [55]. Indeed, it is not known if YAP1 itself acts as a major transcriptional driver of this phenotype or a marker of it [66]. YAP1, a transcriptional key regulator of the Hippo growth signaling pathway, functions as an oncogene and its overexpression induces EMT [67, 68]. YAP1-expressing SCLC cell lines are likely more adherent. Intriguingly, knockdown of YAP1 in many SCLC lines causes morphologic transformation reminiscent of floating growth with concomitant expression patterns of integrin and laminin-mediated cell attachment [69]. Recent analyses revealed that YAP1-regulated repression of the ajuba LIM protein (AJUBA) strongly correlated with shorter overall survival (OS) in SCLC patients [70]. Higher expression levels of YAP1 are associated with worse prognosis and decreased survival with increased chemoresistance [70].

#### Lineage plasticity and transdifferentiation

Epithelial cell types possess diverse functions at steady-state and after injury. The normal lung exhibits a slow cell turnover. However, quiescent stem cells with enormous curative potential arise after epithelial injury. The great diversity of epithelial progenitors implies a high degree of lineage plasticity [71, 72]. In contrast, cancer cell plasticity is referred to the conversion from one committed developmental lineage to a stem cell or another differentiated cell [73, 74]. Intratumoral heterogeneity is a consequence of such plasticity and describes multiple cell populations and gene signatures within a tumor. In contrast, intertumoral heterogeneity refers to heterogeneity across several tumors or patients [75]. Tumor microenvironment (TME)-induced selection pressure drives tumor evolution and consequently associates with therapeutic resistance and metastasis [72, 76]. Furthermore, tumor cell conversion into different histological subtypes is associated with independence from an initial oncogenic driver, selective pressure of therapeutic treatment, and drug resistance [77, 78]. Indeed, LUAD can transdifferentiate into small cell NE cancers (SCNCs) under targeted therapy selection (e.g. tyrosine kinase inhibition (TKI)), (Fig. 1) [1, 78, 79]. Furthermore, transformation into SCLC can occur in anaplastic lymphoma kinase (ALK)-positive lung cancer upon treatment with ALK-inhibitors and in wild-type EGFR or ALK NSCLC following immunotherapy [80, 81]. Although NSCLC and SCLC are different diseases, the histologic transformation from NCSLC into SCLC shares common cells of origin [82, 83]. Indeed, LUAD usually originates from AT2 cells within the peripheral lung regions, and, henceforth acquired resistance to EGFR/ALK-TKI therapy underlies equal molecular mechanisms [82–84]. Emerging evidence indicates that in order to survive unfavorable microenvironments, SCLC tumors transform from NE to non-NE phenotypes driven by the presence of additional molecular alterations or epigenetic changes, signaling pathways, and the TME (Fig. 2) [17, 72, 85]. Thus, a better understanding of the molecular plasticity influencing the functional diversity and corresponding therapeutic response in SCLC remains to be elucidated [73].

#### Subtype switching

**Fig. 3:**
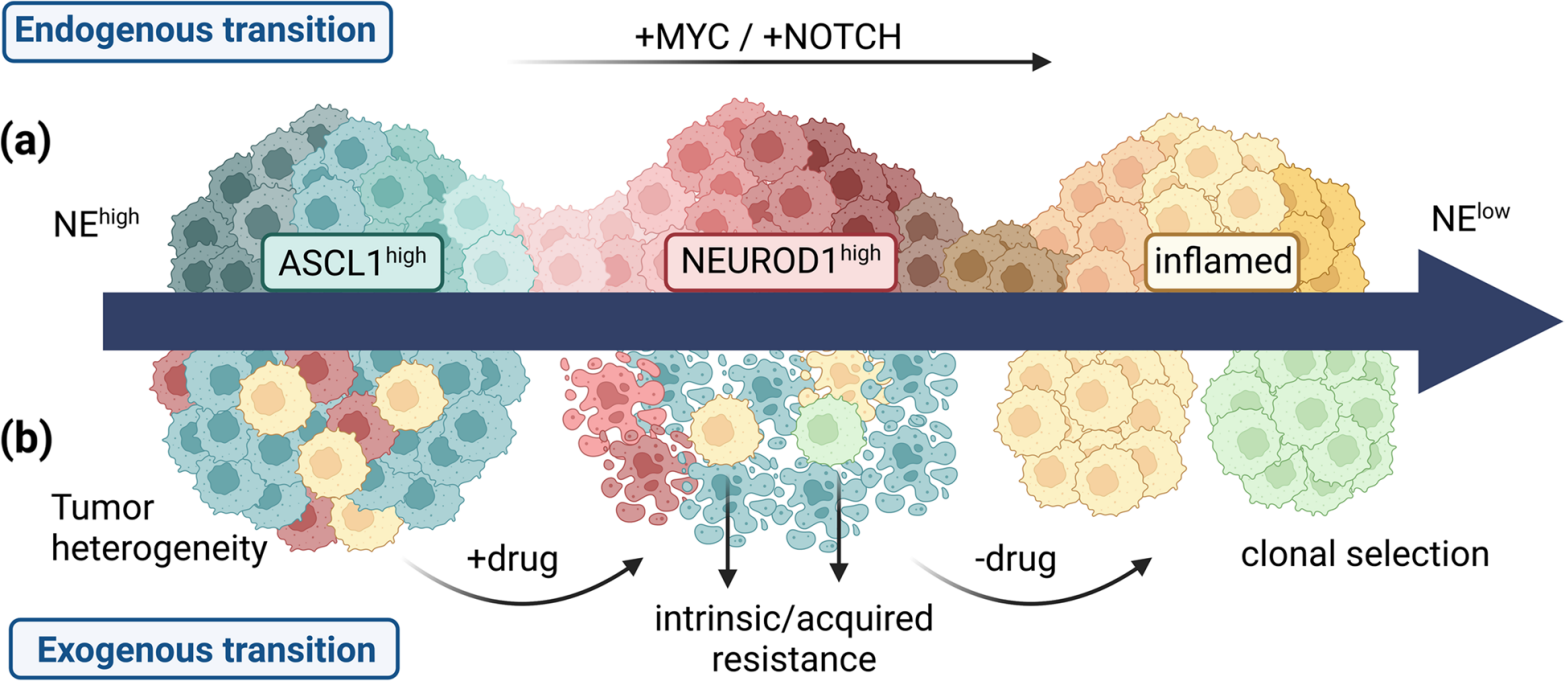
Intratumoral heterogeneity of SCLC based on endogenous or exogeneous influences. **a** Intratumoral subtype switching is facilitated by enriched MYC expressions and/or increased Notch activity. This endogenous transition is assumed to proceed from NE high tumors to non-NE phenotypes. Hence, ASCL1-driven SCLCs transform into NEUROD1 and, ultimately, into YAP1-high tumors. **b** Exogeneous subtype transition is caused by systemic therapy, leading to selection of subclones showing intrinsic or acquired resistance. Disease recurrence accompanied by chemoresistance represents a hallmark of SCLC. Created with BioRender.com. NE - Neuroendocrine; ASCL1 - Achaete-scute homologue 1; NEUROD1 - Neurogenic differentiation factor 1; MYC - MYC proto-oncogene

Notch signaling is generally suppressed in NE SCLC which may induce a transition from NE to non-NE cells. Recent studies demonstrated that MYC activates Notch signaling, driving the temporal evolution of SCLC heterogeneity by conducting the sequential conversion of SCLC tumor cells from an ASCL1- to a NEUROD1- and, ultimately, to a non-NE (YAP1) state (Fig. 3a) [47, 86–88]. Similarly, Notch activity induces a non-NE fate in SCLC models associated with MYC-L [24, 89]. MYC-expressing, Notch-active cells display variant morphology and expression of NEUROD1 or YAP1. However, MYC-negative SCLCs with Notch activity did not exhibit the aforementioned features [86]. Conclusively, Notch activity alone is insufficient to promote the development of SCLC-N and SCLC-Y subtypes. It is more likely that MYC and Notch work together to drive the progression of SCLC.

SCLC tumors may exert bidirectional cell state transitions. Most tumors consist of multiple SCLC cell types, and intratumoral subtypes may collaborate to promote tumorigenicity and cellular diversity. Based on fluorescent promoter sequences of ASCL1, NEUROD1, or YAP1 to directly measure the switching of SCLC subtypes, it was shown that each TF was associated with distinct morphological characteristics and localization within the tumors [90]. Dual reporter experiments showed minimal coexpression of TFs, indicating mutually exclusive cell states. However, transition rates varied among the different state pairs and were influenced by factors such as cell density. Accordingly, the conversion into an ASCL1-dominant state following increased cellularity indicated that cell–cell signaling may impact transition rates [90]. Extensive contacts between YAP1-expressing cells and NE-high cells displayed commensal niche-like interactions. Moreover, phenotypic switching between therapy-sensitive (i.e., NE) and therapy-resistant states (i.e., mesenchymal or neural) has been associated with underlying persistence of SCLC despite initial responses to chemotherapy (Fig. 3b) [90]. A recent study demonstrated that immune-cold SCLCs (such as SCLC-A) lost their NE signature upon AURKA inhibition, resulting in a transition towards an inflamed phenotype. Thus, combined PD-L1 and AURKA therapy displayed durable efficacy in vivo [91]. These data indicate a high fluctuation of subtype plasticity in SCLC tumors.

#### Mechanisms of resistance

Several studies have added to the understanding of molecular mechanisms of acquired resistance in SCLC in the past years. When DNA damage occurs, caused for example by extrinsic damaging agents, tumor cells need to maintain the integrity of the genome for further cell proliferation and survival [92]. Consequently, several research groups hypothesized that contributors to drug resistance are primarily associated with altered DNA damage repair [93]. As such, poly(ADP-ribose) polymerase (PARP) proteins are commonly activated in response to DNA damage caused by platinum and UV radiation, thus, playing a pivotal role in base excision repair (BER) and nucleotide excision repair (NER) [94]. The growing interest in DNA damage response (DDR) is driven by the discovery that aggressive tumors such as SCLC exhibit alterations in DDR pathways. Preclinical research has shown that inhibiting DDR proteins like PARP enhances the anti-tumor immune response facilitated by PD-L1 inhibition through T cell-mediated effects [95, 96]. Accordingly, the proposed synergistic response of combined ICI and PARPi therapy has prompted numerous clinical trials. In SCLC, a phase II trial (NCT04701307) evaluated the efficacy of combination therapy including the anti-PD-1 monoclonal antibody dostarlimab and the PARP inhibitor niraparib in pre-treated extensive-stage patients [97]. Although the interim futility criteria were not met in NE carcinomas, SCLC patients exhibited durable disease control [97]. A recent study focused on the high-mobility group box protein B1 (HMGB1) and its strong correlation with chemoresistance in SCLC. The study illustrated how HMGB1 is able to initiate PARP1 self-modification, facilitating its interaction with microtubule-associated protein/light-chain 3 (LC3) and promoting nucleophagy which contributes to chemoresistance in SCLC [98]. Another study identified YES1 as a novel targetable oncogene that drives SCLC proliferation and metastasis, showing significant antitumor efficacy in organoid models as well as in cell- and patient-derived xenografts (CDX/PDX). This study also demonstrated alterations in the DDR process following treatment with the YES1 inhibitor CH6953755 or dasatinib [99].

A prevalent form of chemoresistance observed in numerous cancers is multidrug resistance (MDR) that is frequently mediated by P-glycoprotein (MDR1) and MDR-related proteins (MRP1 and MRP2) via ATP-dependent efflux [100]. Studies have reported that elevated expression of MDR1 and MRP1 in human samples and xenografts is associated with a poor prognosis and increased chemoresistance [101, 102]. Numerous anti-tumor drugs used to treat SCLC effectively induce DNA damage, thereby affecting the viability of rapidly proliferating tumor cells. Glutathione (GSH), a thiol-containing molecule, enhances the repair of DNA damage and prevents apoptosis [103]. Furthermore, ferroptosis, a form of iron-dependent cell death, is triggered by the deactivation of cellular antioxidant defenses dependent on GSH. This leads to the iron-dependent accumulation of harmful lipid reactive oxygen species (ROS). The activation of ferroptosis has been shown to impede tumor growth and play a role in the development of chemotherapy resistance [104].

#### Cancer stem cells and corresponding signaling pathways

CSCs, also referred to as tumor-initiating cells, constitute a small population of malignant cells with unlimited proliferative potential. They show high capacity for self-renewal, metastatic dispersal, and therapeutic resistance [105]. The cell of origin of CSCs remains unknown, but they phenotypically and functionally resemble normal stem cells of the same tissue [106]. There is growing evidence that CSCs originate from normal cells by gaining stem cell-like characteristics, which is mostly due to EMT [107–109]. EMT is fundamental for physiological processes including epithelial generation during embryogenesis, organogenesis, and wound healing [110]. Moreover, EMT influences the pathophysiology of fibrosis and various malignant transformations. It refers to the dedifferentiation of stationary epithelial cells into motile mesenchymal cells, thereby promoting cancer progression. Conversely, mesenchymal-to-epithelial transition (MET) describes the reverse process. Both EMT and MET endow cell migration to distant organs, induce pluripotency and CSC properties, prevent apoptosis and senescence, and contribute to immunosuppression [111, 112]. Stem cell signaling facilitates intratumoral heterogeneity and promotes EMT phenotypes in SCLC. CSCs may also arise from non-malignant, dysregulated progenitor cells, which ultimately results in aberrant plasticity [113]. Importantly, CSCs are characterized by particular cell surface markers including CD133, CD44, CD117, CD90, CD166, and epithelial cell adhesion molecule (EpCAM) in NSCLC. Likewise, PODXL-1, PTCH, and CD87, as well as aldehyde dehydrogenase (ALDH) enzymatic activity are associated with SCLC [114–117]. Notably, the identification of CSCs from human solid tumors remains challenging due to heterogeneous cell populations that may substantively confirm stem-cell-like properties [118].

The dysregulation of signaling pathways may induce CSCs occurrence and tumorigenesis [116]. Developmental signaling pathways (including the Hippo, Notch, Hedgehog (HH), and Wnt/β-catenin pathways) are frequently altered in lung CSCs [119, 120].

**Fig. 4:**
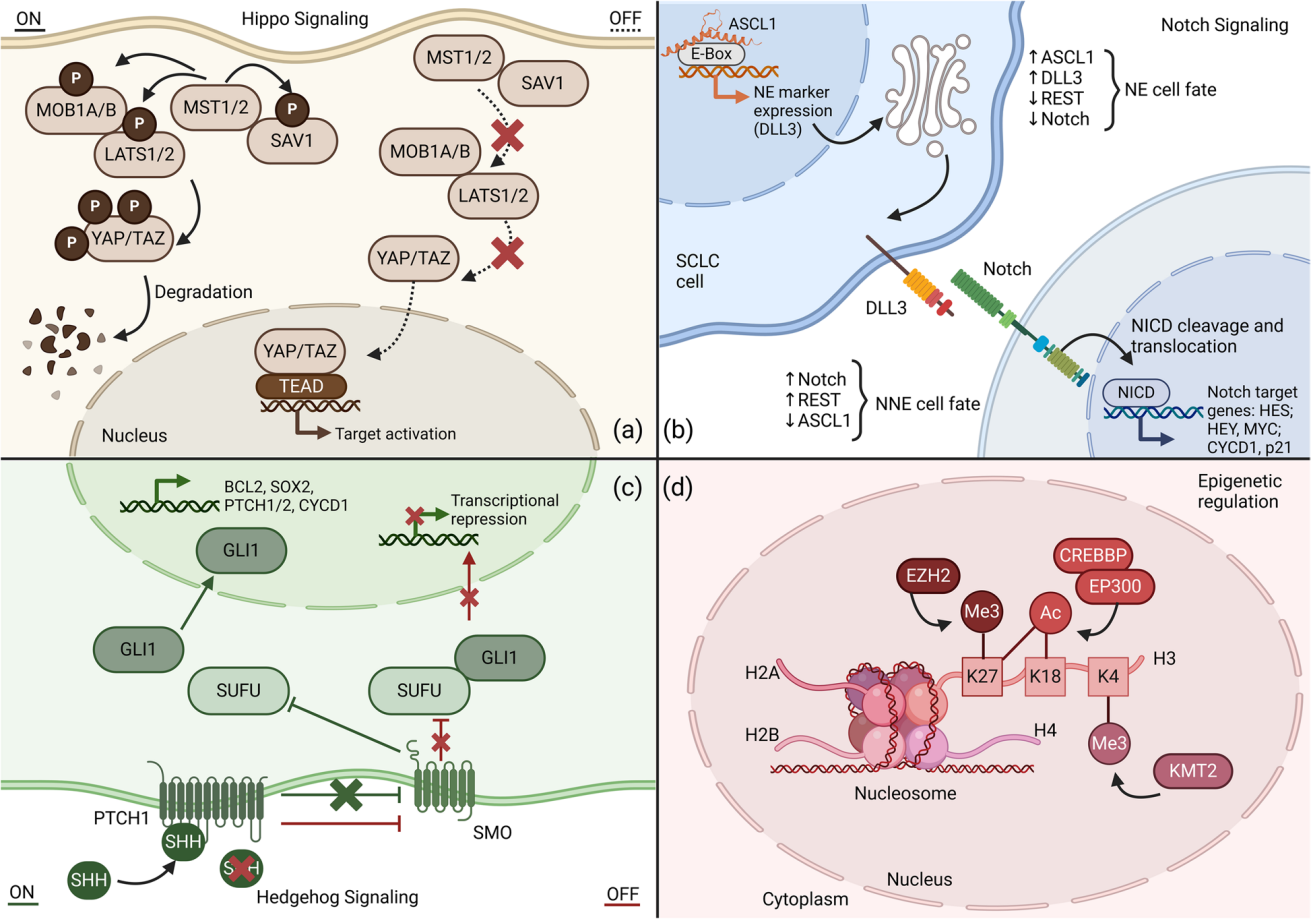
Frequently altered signaling pathways in SCLC. **a** The Hippo signaling pathway discriminates between active (ON) and inactive (OFF) states. When Hippo signaling is on, the phosphorylation of SAV1 is mediated by MST1/2, both further activating MOB1A/B and LATS1/2 via phosphorylation. Ultimately, the YAP/TAZ complex is phosphorylated and degraded, resulting in transcriptional repression. Or in contrast, when Hippo signaling is off, the phosphorylation cascade does not take place. The YAP/TAZ complex translocates into the nucleus and interacts with TEAD, leading to transcriptional activation. **b** The Notch signaling pathway is mediated between signal-sending and signal-receiving cells via interaction of the Notch receptor and a Notch ligand (DLL or JAG). Consecutively, the Notch receptor is cleaved; NICD translocates into the nucleus and activates the transcription of target genes in signal-receiving cells (e.g. HES, HEY, and MYC). ASCL1 is categorized as a master regulator of NE differentiation via high expression of the non-functional DLL3 at the golgi apparatus. DLL3 acts as a dominant-negative inhibitor of Notch signaling and orchestrates the degradation of other Notch members. HES and HEY family members encode transcriptional repressors of ASCL1. Notch negatively regulates NE differentiation in SCLC. High ASCL1 expression levels significantly correlate with NE differentiation. **c** Hedgehog signaling is activated by the binding of SHH to PTCH1. This leads to the shift of inhibitory activity towards SUFU (*green path*). Subsequently, the GLI1 monomer translocates into the nucleus and promotes gene transcription. When PTCH1 exerts its inhibitory effects against SMO, SUFU and GLI1 build a complex which inactivates the expression of HH target genes (*red path*). **d** Epigenetic reprogramming plays an instrumental role in SCLC via methylation / acetylation of DNA/histones. Key chromatin modifiers are EZH2, the CREBBP/EP300 complex, and the KMT2 family proteins which each target the amino acids K27, K18, or K4 of histone 3, respectively. Created with BioRender.com. ASCL1 - Achaete-scute homologue 1; DLL3 - Delta-like protein 3; REST - RE1-silencing transcription factor; HES - HES family BHLH transcription factor 1; HEY - HES-related repressor protein 1; MYC - MYC proto-oncogene; CYCD1 - Cyclin D1; p21 - Cyclin dependent kinase inhibitor 1A; MOB1A/B - MOB kinase activator 1A/B; MST1/2 - Mammalian sterile 20-like kinases 1 and 2; LATS1/2 - Large tumor suppressor kinase 1/2; SAV1 - Salvador homolog 1; YAP1 - Yes-associated protein 1; TAZ - Tafazzin family protein; TEAD - TEA domain transcription factor; EZH2 - Enhancer of zeste homolog 2; Me - Methylation; Ac - Acetylation; CREBBP - CREB binding protein; EP300 - E1A binding protein P300; H2A/2B/3/4 - Histone 2A/2B/3/4; KMT2 - Lysine methyltransferase 2; SHH - Sonic hedgehog; GLI1 - GLI family zinc finger 1; SUFU - SUFU negative regulator of hedgehog signaling; PTCH1/2 - Patched 1; SMO - Smoothened, frizzled class receptor; BCL2 - B-cell lymphoma 2; SOX2 - SRY-box transcription factor 2

The Hippo signaling pathway balances proliferative and apoptotic capacities of cells by initiating the mammalian sterile 20-like kinases 1 and 2 (MST1/MST2) with the adaptor protein salvador homolog 1 (SAV1) and the large tumor suppressor kinase 1/2 (LATS1/LATS2) with the MOB kinase activator 1 (MOB1) as an adaptor protein (Fig. 4a) [121]. During pathway activation, consecutive phosphorylation events are mediated by these kinases [122]. Because YAP1 and the transcriptional coactivator with PDZ-binding motif (TAZ, a homologue to YAP1) cannot bind to the DNA directly, they must interact with DNA-binding TFs. The TEA domain transcription factor (TEAD) family regulates target genes when Hippo signaling is inactivated [123]. Indeed, altered Hippo signaling causes increased YAP1 and/or TAZ activity, leading to the extension of CSC populations. This contributes to solid tumorigenesis, cancer progression, and chemoresistance, primarily in non-NE SCLC [124].

Notch signaling regulates stemness with pro-tumorigenic or tumor suppressive activity in SCLC. Notch negatively regulates NE-differentiation and is suppressed in the majority of SCLCs [125]. Notch signaling is activated by the binding of DLL1/3/4 or Jagged-like ligands (JLL1/2) to their receptors (Notch 1–4) leading to a conformational change. Additionally, Notch receptor cleavage is promoted when γ-secretase releases the Notch intracellular domain (NICD) from the cell membrane (Fig. 4b) [126, 127]. NICD translocates into the nucleus where it interacts with the DNA and induces the transcription of the transcriptional regulators HES1, and hairy and enhancer of split-related protein 1 (HEY1) [128]. HES1 and HEY1 act as transcriptional repressors of ASCL1, which are themselves activators of the expression of Notch ligands [129]. The non-functioning ligand DLL3 is expressed accordantly with ASCL1 and acts as a dominant-negative inhibitor of Notch signaling [130]. Endogenous activation of Notch signaling accompanied by loss of NE differentiation results in a NE to non-NE fate switch, which is mediated partly by the RE1 silencing transcription (REST) co-repressor (Fig. 4b) [89].

The HH pathway participates in embryonic and early hematopoietic stem cells. Aberrant HH signaling has been linked to various solid and hematological cancers [131–133]. The paracrine HH ligands include sonic hedgehog (SHH), indian hedgehog (IHH), and desert hedgehog (DHH). These isoforms bind to the transmembrane receptors patched 1 (PTCH1) and PTCH2, and mitigate an inhibitory effect on smoothened (SMO) [134]. Subsequently, the initiated signaling cascade leads to the inhibition of suppressor of fused (SUFU) and to the activation of the glioma-associated oncogene family (*GLI1, GLI2,* and *GLI3*). Consequently, HH target genes, including *SOX2*, are actively transcribed (Fig. 4c) [135]. Determining the role of these pathways in cancer and their intracellular crosstalk is a promising area for novel targeted therapies [116].

#### Epigenetic modifications

Epigenetic changes involve post-transcriptional DNA modifications which have profound effects on gene expression. Moreover, epigenetic processes, which include DNA methylation, histone modification, and chromatin remodeling, lead heritable changes in gene expression and may control cell identity [136]. Important epigenetically silenced genes in SCLC include *NEUROD1*, *REST*, TF2 (*TCF2/HNF1B*), retinoic acid receptor beta (*RARB*), and *BCL2*. Further genes located in the critical chromosomal region 3p include *RASSF1A* and enhancer of zeste homologue 2 (*EZH2*) [137, 138]. EZH2 acts as a master regulator of transcription and affects DNA methylation via chromatin modification and activation of DNA methyltransferases (DNMTs) by targeting the CpG islands (Fig. 4d) [139]. The upregulation of EZH2 correlates with poor prognosis in SCLCs [140]. EZH2 overexpression leads to epigenetic silencing of transforming growth factor-β (TGF-β) receptor type 2 expression and suppression of apoptosis. This results in altered DNA methylation which promotes SCLC progression by suppressing the TGF-β-Smad-ASCL1 pathway [141]. Further chromatin modifiers comprise the histone acetyltransferase (HAT) genes CREB-binding protein (CREBBP or CBP) and EP300 as well as the epigenetic readers encoded by *MLL* and *MLL4* (also known as histone methyltransferase genes *KMT2A* and *KMT2D*) (Fig. 4d) [142]. Lysine Demethylase 6A (KDM6A) which shows a high affinity to KMT2A binding, is an epigenetic regulator affecting chromatin accessibility, thereby controlling ASCL1-to-NEUROD1 subtype switching [143]. CREBBP is a transcriptional coactivator of the Wnt/β-catenin signaling pathway and functions as a HAT [144]. *CREBBP* is commonly mutated in human SCLC and shows homology to EP300, another HAT. Deletion in CRB/EP300 reduces histone acetylation and transcription of cellular adhesion genes while driving tumorigenesis [44, 145]. These effects can be partially restored by histone deacetylase (HDAC) inhibition [44, 146].

The bromodomain and extraterminal domain (BET) family regulates MYC expression and amplification in SCLC [147]. Since chromatin modifiers and TFs conquer the super-enhancer role, inhibition of super-enhanced oncogenes such as *MYC, BCL2, ELF3*, or *NFIB* represents a potential therapeutic strategy [148, 149]. Additionally, the Schlafen family of proteins (SLFN11) controls cell proliferation and induces immune responses [150]. High expression of SLFN11 correlates with response to chemotherapy including cisplatin and PARP inhibitors, while SLFN11 downregulation is associated with cisplatin resistance [151]. Moreover, the overexpression of EZH2 mediates chemotherapeutic resistance by downregulating SLFN11 through histone modification and methylation in a subset of SCLCs [150].

### Experimental models in SCLC

**Fig. 5:**
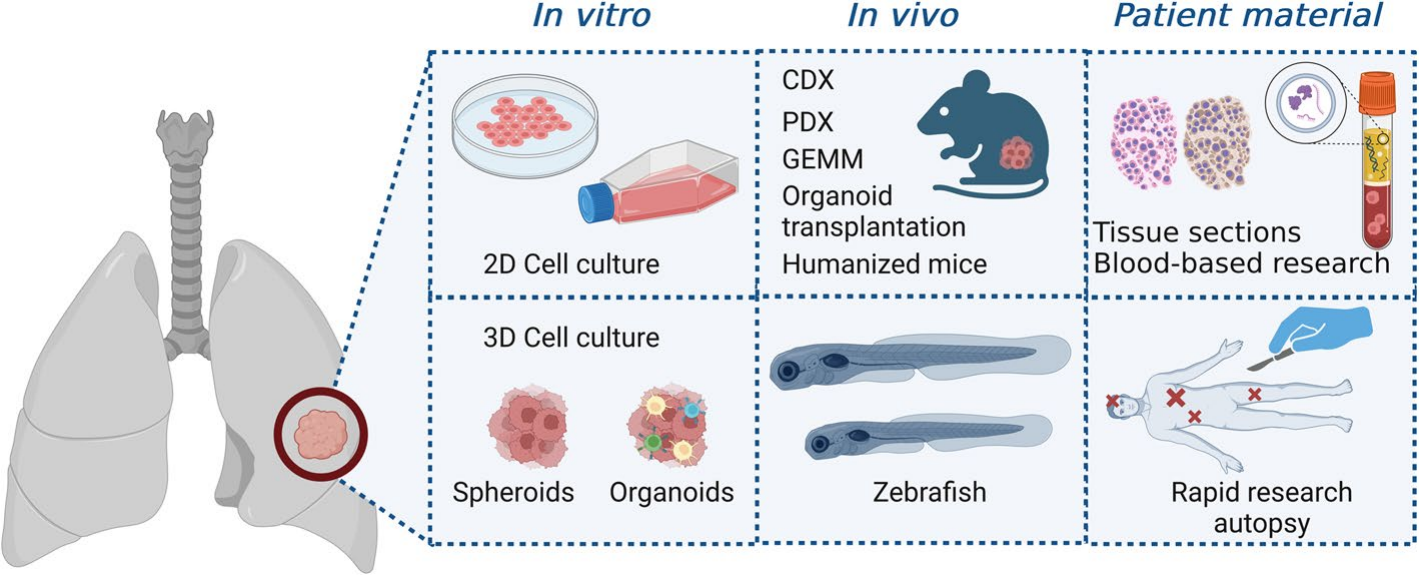
Fundamental preclinical models for SCLC research. Preclinical research commonly relies on well-established assays based on two-dimensional (2D) or three-dimensional (3D) cell culture. Different cell compositions (cell lines, circulating tumor cells, tissues) can be furthermore examined in vivo via subcutaneous or orthotopic transplantation. Hence, CDX or PDX xenotransplantation mimic human disease regardless of immune microenvironment. The genetically engineered mouse model (GEMM) displays a platform to investigate tumor initiation and tumorigenesis of SCLC via genetic knock-out. In contrast, humanized mice present intact immunologic features that may be exploited to investigate the interaction between SCLC tumors and their microenvironment. Zebrafish larvae are frequently utilized to study the dynamic invasion of tumor cells in vivo or evaluate cost-effective chemical screenings. Sampling of representative tissue samples is rather rare in SCLC patients due to infrequently performed surgery. Rapid research autopsies should be used with greater frequency in SCLC research. Created with BioRender.com. CDX - Cell-derived xenograft; PDX – Patient-derived xenograft; GEMM – Genetically engineered mouse model

More than two-thirds of SCLC patients suffer from metastatic tumor manifestation, which excludes surgical resection from the list of potential treatment options [152]. Moreover, moderate success from surgical resection has hindered tumor biobanking in SCLC [153]. Preclinical research in SCLC often relies on classical methods based on cell culture and animal models (Fig. 5).

#### Cell lines

The first human SCLC cell line was established from a metastatic lymph node in 1971 [154]. This original classification of SCLC was characterized by floating culture properties. Since then, multiple SCLC cell lines have been established and have determined the beginning of preclinical research [155, 156]. Gazdar et al. first underscored the importance of human lung cancer cell lines by highlighting the presence of more than 8,000 citations on past cell line studies [157]. Nowadays, the number of citations in the PubMed database attributable to human lung cancer cell lines has potentiated to more than 60,000. Although SCLC shows rapid tumor growth, the establishment of cell cultures is rather difficult as tumor cells do not entirely harbor adherent properties, but commonly grow in suspension and require special media. Since cell lines consist of one clone, they only represent a subpopulation of the neoplasm and do not consider the high intratumoral heterogeneity. Nonetheless, established SCLC cell lines are capable of retaining their characteristic NE features in vitro [158].

Human SCLC cell lines represent a fundamental tool for investigating novel therapeutic agents. Accordingly, multiple treatment approaches have been proposed to overcome drug resistance including the inhibition of exportin-1 (*XPO1*). Decreased *XPO1* levels sensitized SCLC to cytotoxic therapy in vitro [159]. This novel target was identified through SCLC cell line screening using CRISPR-Cas9. Furthermore, the XPO1 inhibitor selinexor acted synergistically with cisplatin or irinotecan in vitro and reduced tumor formation in vivo [159]. Several phase I/II trials evaluated the combination of selinexor with chemotherapy in metastatic solid tumors (NCT02419495, NCT04256707) [160, 161]. Overall, the combination showed acceptable tolerability and clinical activity, but superior efficacy has to be further investigated in tumor entities where CHT remains the standard-of-care therapy [161]. Conclusively, pre-clinical results often coincide with potential novel clinical approaches and vice versa.

The SCLC-CellMiner database which comprehensively comprises data of 118 patient-derived SCLC cell lines has been recently established [162]. This dataset provides in-depth information about genomic signatures, methylome and transcriptome data as well as drug sensitivity profiles. Accordingly, results from SCLC cell lines are reportedly reproducible and confirm the classification of SCLC molecular subtypes. Besides the commonly known TFs, these datasets have also been linked with prominent signaling pathways including Notch and Hippo [162]. Similarly, proteomic results obtained from human-derived SCLC cell lines and patient-derived samples demonstrated comparable results [163, 164]. Another useful database for advancing SCLC research is the Cancer Dependency Map (DepMap) portal. In general, DepMap aids in elucidating cancer-specific therapeutic vulnerabilities and identifying novel biomarkers across hundreds of cancer cell lines by providing open access to key cancer dependencies. This tool has particularly been used in SCLC to decipher the TF-driven signatures and corresponding vulnerabilities of SCLC subtypes. In SCLC-A, interactions between ASCL1, NKX2-1 (TTF1), and prospero homeobox protein 1 (PROX1) have been reported to modulate Notch signaling and genes involved in cell cycle regulation [165]. DepMap calculations revealed mutually exclusive correlations between high POU2F3 and C11orf53 (POU2AF2) or COLCA2 (POU2AF3) expression levels in SCLC cell lines and patient samples, hence emphasizing a unique biological association with tuft cell-like properties [166, 167].

#### Organoids

*In-vitro* assays in a three-dimensional (3D) setting or multicellular organoids represent emerging techniques in cancer research. In contrast to normal floating aggregates in 2D culture, spheroids are defined as simple 3D cell aggregates frequently consisting of one cell type. Organoids, in turn, represent complex clusters of multiple cell types resembling the original tissue. Human stem cells derived from lungs or other organs were recently cultured in a 3D setting [168, 169]. Once embedded, stem cells start to self-organize in extracellular matrix such as collagen, start proliferating and differentiating [170]. Occasionally, malignant cells manage to organize or maintain 3D growth in vitro. The established organoids maintain the architecture of parental tumor tissues. Even after longitudinal culture, organoids perpetuate genetic patterns and morphological characteristics [171]. Cancer organoids represent suitable in vitro tools that are less time-consuming and cost-intensive. However, organoids require additional nutrients and are not yet capable of self-constructing into organ- or tissue-specific functional structures and show limited long-term differentiation [172].

Although SCLC is highly heterogeneous, established SCLC organoids sustain the characteristic histological properties including small cell morphology, expression of NE markers, and genetic programs compared to original tumor tissues [173]. Intriguingly, the intratumoral heterogeneity of SCLC remains present in organoid-based assays which is reflected by the differential sensitivity to chemotherapy [171]. Furthermore, organoids are popular tools to investigate the complex interplay between tumor cells and the multifaceted microenvironment. Orthotopically transplanted SCLC organoids resulted in metastatic dissemination, which was associated with epigenetic changes including KMT2C loss [174]. KMT2C is a histone H3 lysine 4 methyltransferase frequently altered in ES-SCLC patients [142]. CRISPR editing of the key driver genes in normal lung tissue of SCLC, namely *TP53*, *RB1,* and *C-MYC* ultimately lead to organoid formation [174]. Hence, the primary lesion derived from such organoids mimics human disease more thoroughly compared to GEMM where multiple lesions are common [174]. Intriguingly, morphological structures confirmed SCLC origin along with disease-specific markers including ASCL1, CHGA, or SYP.

Organoids obtained from patient tissues preserve the histological subtypes of lung cancers [175]. Moreover, organoids maintain cancer-characteristic mutations such as *EGFR*, *BRCA2,* or *MET*. Regarding SCLC, the established organoids showed typical morphology of small round cells characterized by high nuclear to cytoplasmic ratio and granular chromatin. Accordingly, several NE markers were present in SCLC organoids including NCAM1, SYN, and TTF-1 [175]. Patient-derived tumor organoids display a useful tool for therapeutic screening [175]. Of note, half-maximal inhibitory concentrations (IC_50_) of olaparib (PARP inhibitor), erlotinib (EGFR inhibitor), or crizotinib (MET inhibitor) were shown to correlate with corresponding genetic alterations. Olaparib was most efficient in BRCA2-deficient samples, the TKI erlotinib showed differential responses in *EGFR*-mutant organoids, and crizotinib effectively targeted organoids harboring *c-MET* alterations [175]. Altogether, organoid-based preclinical research represents an emerging and reliable tool for SCLC research.

#### Mouse xenograft models

Both intra- and intertumoral heterogeneity can be evaluated by IHC staining of SCLC tissue specimens. However, preclinical in vitro models such as cell lines do not exhibit molecular or phenotypical heterogeneity because they represent a single clone of the original tumor. In contrast, in vivo models such as GEMMs or PDXs enable the display of tumor heterogeneity and plasticity [62]. Whole-exome sequencing (WES) revealed the enduring preservation of somatic mutations in PDXs compared to primary tumors. Moreover, the genomic and transcriptional signatures of sub-cultured PDXs closely mirror those of the original PDX [176]. Serial xenografts derived from circulating tumor cells (ctCDX) at different time points of a SCLC patient accurately matched the changing drug sensitivities of the patient's disease. This data underscores the promising translational potential of employing this preclinical approach [176].

CtCDXs and PDXs are commonly used to elucidate the genetic exceptionalism of SCLC. Recently, multi-omics analyses from both CDX and PDX of human SCLCs highlighted the genomic landscape of three SCLC subtypes driven by ASCL1, NEUROD1, and POU2F3 [177]. The xenografts preserved NE/non-NE characteristics, hence corroborating their preclinical applicability. However, YAP1 expression has scarcely been detected in clinical specimens or xenografts [55, 61]. In line with previous data, POU2F3-driven PDXs were strongly associated with high MYC levels [177]. MYC along with stemness-associated markers CD44 and SOX2 are positively regulated upon ligand–independent EPHA2 (erythropoietin-producing hepatocellular A2) activation, which strongly increases stemness and chemoresistance in SCLC [178]. CSCs show high cell plasticity and harbor distinct features to reversibly change their epithelial or mesenchymal state, thereby embodying key players in drug resistance and invasiveness [107]. EPHA2 activates PRMT1 (protein arginine methyltransferase 1), which in turn methylates SOX2 to promote stemness and chemoresistance. The inhibition of EPHA2 in a SCLC PDX model revealed synergistic interactions with chemotherapy following tumor regression [178]. EPHA2 inhibition is under current clinical investigation, primarily in advanced solid tumors (NCT01591356, NCT04180371). However, the efficacy of targeted therapy against EPHA2 in SCLC remains to be elucidated.

In mice bearing genetically modified SCLC tumors, metastatic lesions were primarily located in the liver and lymph nodes which is in line with common metastatic sites of SCLC patients [179]. Omics-based analysis using single-cell RNA sequencing suggested epigenetic differences between primary and metastatic tumors. The gene locus of *KMT2C* was transcriptionally repressed in metastatic lesions, presenting a novel epigenetic program of SCLC metastasis [174].

Mouse xenograft models have been used to explore chemoresistance in SCLC via longitudinal intermittent chemotherapy imitating clinical applications [180]. One major finding comprises the metabolic reprogramming of the mevalonate-geranyl–geranyl-diphosphate (MVA-GGPP) pathway in chemoresistant SCLCs [180]. Oxidative stress accumulates upon statin treatment and induces apoptosis via the GGPP synthase 1-RAB protein axis. GG-Rab proteins regulate Notch processing and are linked to differential responses in SCLC subtypes [181]. However, GGPS1 expression was associated with poor survival in SCLC patients [180]. Dual statin- and chemotherapy of three relapsed patients resulted in durable responses, highlighting the potential of targeting metabolic changes in SCLC [180].

#### Genetically engineered mouse models

Animal models are expensive, resource-intensive, and time-consuming [175]. However, data from mouse models data have been fundamental for the most remarkable discovery in SCLC. The recent proposition of SCLC molecular subtypes partly relies on GEMM data in combination with results from human tumor samples [4].

GEMMs are generated via alteration of target driver genes that are functionally conducive for context-dependent tumorigenesis. The first SCLC GEMM was established by Anton Berns in 2003 [182]. Using the Cre/LoxP-mediated recombination system, both TS genes *TP53* (floxed exons 2–10) and *RB1* (floxed exon 19) were inactivated, causing small cell NE tumors [183]. The murine SCLCs highly resembled human disease. Manipulation of lung epithelial cells induced malignant transformation only in NE and AT2 cells upon *Tp53* and *Rb1* disruption, thereby confirming the SCLC cells of origin [9]. Beyond the Cre recombinase and adenoviral vector transfection, advanced methods including Lenti-Cre-based mouse models were established. In triple knock-out (TKO) mice with silenced *Tp53*^fl/fl^, *Rb1*^fl/fl^, and *Rb2*^fl/fl^, SCLC tumors evolved within four to six months and acquired NE characteristics accompanied by predominant ASCL1 expression. Barcoding of SCLC tumor clones referring to a panel of 40 candidate genes revealed that *Tsc1* and *Pten* inactivation resulted in increased tumor size, while *Pcna*/*Arid1a* alterations were associated with decreased TMB [184].

In experimental settings, adenoviral Ad-K5cre (Keratin K5 regulated cre recombinase) infection of NMRI-Foxn1^nu/nu^ immunodeficient young mice resulted in the malignant transformation of basal cells into SCLC [185]. Notably, this transformation was characterized by the quadruple loss of floxed TS genes *Rb1*^F/F^, *Tp53*^F/F^, *Pten*^F/F^ and by null retinoblastoma-like protein 1 (*Rbl1*^−/−^) [185]. Since these murine cell lines can be implanted into immunocompetent mice of the same strain, they are particularly useful for immunotherapy studies. Moreover, syngeneic SCLC tumors preserve the transcriptional program of newly established murine cell lines, and display remarkable congruence to human SCLCs [186]. Another advantage of murine SCLC models is their potential to study metastatic progression. Hence, these emerging in vivo models are highly advantageous for studying anti-metastatic and immunotherapeutic approaches [186].

#### Humanized mice

SCLC is known for its propensity to metastasize, leading to infrequent surgical interventions and a paucity of representative samples. The immune system is known to be closely related to tumorigenesis and cancer progression [187]. However, the engraftment of malignant cells or tissues in mice with severe immunodeficiencies has limitations such as xenogeneic immune rejection [188]. Therefore, to exploit the interaction between human tumor cells and the TME, immunocompetent models featuring T-cell chimerism are increasingly being applied in SCLC research [189]. Humanized mice can be generated by injecting human peripheral blood leukocytes or hematopoietic progenitor cells into immunocompromised mice [190, 191]. CDX and PDX tumors of SCLC were successfully grown in humanized mice with a growth rate comparable to immunodeficient mice [192]. Tumor-infiltrating immune cells are sparse in SCLC xenografts, mirroring their incidence in human SCLC tumors. However, human T-cells occur in lymph nodes and spleen. SCLC cells were characterized by the low expression of PD-L1 and MHC class I and II, whereas PD-1 expression was present on the surface of corresponding human T-cells [192]. Although SCLC is associated with high TMB, the response to immune checkpoint inhibition (ICI) is relatively poor. Simultaneous inactivation of WEE1 inhibition and PD-L1 blockade resulted in decreased tumor growth via activation of the STING-TBK1-IRF3 pathway along with cytotoxic T-cell infiltration in immunocompetent SCLC GEMMs [193]. Moreover, WEE1 blockade activated STAT1 signaling, ultimately increasing IFN-γ and PD-L1 expression [193]. Two clinical trials investigating WEE1 inhibition in SCLC were conducted. NCT02688907 was terminated and NCT02593019 was completed, but no further results have been published.

Chimeric antigen T-cell transfer (CAR-T) significantly advances cancer research. Specifically, targeting the AC133 epitope of the stem cell marker CD133 using CAR-T cells showed prolonged survival of humanized mice with orthotopically growing SCLC [194]. Similarly, allogeneic anti-DLL3 CAR-T cells demonstrated high efficacy and safety in SCLC mouse models [195]. CAR-T cells have demonstrated striking results in the treatment of hematologic malignancies [196]. However, their clinical use in SCLC has not yet been fully elucidated. The bispecific T-Cell engager tarlatamab demonstrated manageable safety profiles in an open-label phase I trial (NCT03319940) [197]. Patients with relapsed/refractory SCLC were treated with tarlatamab co-targeting DLL3 and CD3, leading to T-cell-mediated tumor lysis [197]. The median progression-free survival (PFS) was 3.7 months and the OS was 13.2 months, respectively. The study proposed an upfront selection of DLL3-driven SCLCs to obtain superior clinical outcomes [197]. Currently, three clinical trials are focusing on the application of CAR-T cells in SCLC. A phase I study evaluating AMG 119 (CAR-T cell therapy targeting DLL3) has been suspended (NCT03392064). Two other CAR-T-based phase I trials on DLL3 or GD2 (glycolipid tumor antigen) for SCLC are already planned but have not commenced recruitment (NCT05680922, NCT05620342). Hence, upcoming clinical results will need to be reviewed before considering the addition of CAR-T cell therapy to the therapeutic regimen of SCLC.

#### Zebrafish model

The zebrafish has emerged as a valuable model for studying cancer. Advantages comprise their transparency during embryonic development, the large numbers of offspring, cost-effectiveness, and the potential for high-throughput screening. Additionally, the genetic similarity to humans and amenability to genetic engineering make it useful for studying human diseases [198]. However, some organs such as the breast or the lung are not present in zebrafish, and the innate immune system is absent in larval fish < 9 days post fertilization (dpf) and in SCID adults. Additionally, zebrafish require a different temperature for rearing [198]. Recent advances using zebrafish have led to the identification of oncogenic drivers and potential drug targets. As such, cordycepin, an activator of AMP-activated protein kinase (AMPK) and repressor or mTOR signaling was shown to exert significant decrease of proliferation and less brain metastases in SCLC xenografts [199]. RNA-seq analysis revealed that cordycepin affected vitamin D metabolism, lipid transport, and proteolysis in cellular protein catabolic process pathways in the SCLC brain metastasis microenvironment of zebrafish, regulating the expression of key genes such as cyp24a1, apoa1a, and cathepsin L. The anti-brain metastasis effect of cordycepin in SCLC was mediated by reversing the expression of these genes [199]. Overall, though zebrafish represent an expedient asset for advancing our understanding of lung cancer, the implementation of this model in SCLC research has been moderate so far.

#### Analyses of patient material

Since sampling is rare in SCLC patients due to infrequently performed surgery, tissue-based research for the identification of molecular subtypes remains limited. Recent publications comparing whole tissue samples and their corresponding TMA blocks [55] or metastatic lymph nodes [200] demonstrated a significant overlap of TF expression. Moreover, human tissues differentially expressed immune markers including CD47 and PD-L1. IHC stainings revealed a high distribution of CD47 in surgically resected SCLC samples (84.6%) [201]. Although PD-L1 inhibition plus chemotherapy has been approved for the treatment of ES-SCLC patients, the protein expression in tissue samples is moderate (9.6%) [201]. However, the tumor-associated stromal tissue revealed higher PD-L1 distribution in 59.6%. Of note, better OS significantly correlated with high stromal PD-L1 [201]. In non-NE tumors, immune phenotypes displayed high CD8 + T-cell infiltration, supporting the inflamed SCLC subtype [202]. NE subtypes furthermore displayed high MYC levels, whereas non-NE SCLC tumors were frequently associated with increased L-MYC expression [202].

Rapid research autopsy (RRA) is another method to obtain patient material where tumor specimens are collected within few hours post-mortem. Our group recently comprehensively highlighted the importance of RRA in SCLC [203]. The implementation of RRA may serve as a promising platform for obtaining SCLC specimens and for delineating differences between primary/metastatic neoplasms and molecular subtypes.

Current aspirations in SCLC are the identification of blood-based biomarkers and the characterization of the molecular subtypes based on blood. Next-generation sequencing (NGS) of cell-free DNA (cfDNA) validated aberrant genes and copy number variations commonly altered in SCLC [204]. Such alterations included *TP53* mutations, *MYC* amplifications as well as inactivating mutations in Notch family genes, demonstrating the great potential of liquid biopsy in SCLC [205]. Furthermore, results of methylation profiling comparing cfDNA and patient-derived tissues revealed a strong correlation [206]. The SCLC molecular subtypes were successfully detected and portrayed similar NE and non-NE backgrounds [206]. DNA methylation profiling has also been reported recently to allow molecular subtyping of both SCLC tissue and blood samples [207]. Additionally, the differentiation of lung cancer types including NSCLC and SCLC based on liquid biopsy demonstrated 25% specificity and 40% sensitivity [208]. cfDNA and single-cell sequencing of circulating tumor DNA (ctDNA) and CTCs hold great potential for subtype assessment and biomarker-directed therapy. The RASTEN-A randomized trial reported a steady decline of CTCs in SCLC patients during and post-treatment [209]. Furthermore, the baseline number of detectable CTCs significantly correlated with worse survival [209].

### Emerging therapies

#### Immunotherapy in SCLC

Unlike NSCLC, where improvements in personalized therapy and immunotherapy have revolutionized the therapeutic armamentarium, SCLC outcome has remained moderate upon ICI administration. Immunomonotherapy has not been considered in the first-line treatment of SCLC due to rapid disease progression [210]. However, combinational strategies using chemotherapy and PD-L1 inhibition gained approval in 2019/2020 in the US and EU for the initial treatment of SCLC [211, 212]. Due to increased rates in TMB and common paraneoplastic syndromes (PNSs) in approximately 10% of patients, SCLC is perceived as highly immunogenic [213, 214]. Autoimmune responses occur against specific antigens pathologically expressed by SCLC cells, leading to specific antibody production [213, 215]. SCLC patients that present with anti-neuronal nuclear antibody (ANNA)-caused PNS show improved survival outcomes following chemotherapy [216, 217]. However, cancer patients treated with anti-PD-L1 or anti-PD-1 exhibited worsened PNS or even developed PNSs [218].

Preclinical studies advanced the identification of synergistic drug interactions of chemo-immunotherapy. As the efficacy of ICIs is frequently associated with high TMB, a strong pharmacological rationale for combination therapy has been arisen [219]. The therapeutic transformation into SCLC immune-oasis was shown to ameliorate response to immunotherapy [216]. Induction of an immunosuppressive milieu can be facilitated through negative regulation of MHC antigen expression or by diminishing the function of cytotoxic CD8 + T-cells [220]. Combined chemo-radiotherapy shows immunomodulatory effects, hence, inducing immunogenic tumor cell death via apoptotic cell phagocytosis of antigen-presenting cells (APC) or cross-presentation of cancer neoantigens to T-cells [210]. Dual targeting of ICIs with CHT or radiotherapy can efficiently restore the anti-tumor activity of the immune system, hence showing their characteristic and synergistic drug interactions [210]. Augmented expression levels of both PD-1 and PD-L1 have been ascertained in several lung cancer cell lines/tissues, particularly in cisplatin-resistant SCLC cell lines [221]. Preclinical data proposed improved chemosensitization when targeting the PD1/PD-L1 axis in SCLC [221].

#### Subsequent-line therapy and experimental therapeutic concepts

In the event of recurrence or progression, a distinction should be made between local or systemic recurrence. In the rare case of a local recurrence, radiotherapy or even surgical resection may be considered. However, in this case patients must be well selected and distant metastases must be precisely excluded. Subsequent-line treatment options for SCLC patients who progress during first-line chemo-immunotherapy are scarce. Re-challenging with the original regimen or a similar platinum‐based compound is recommended if relapse occurs after six months from initial therapy and can be considered if the relapse-free period is between three and six months [5, 222]. For cases when re-challenging is not feasible, the FDA and EMA approved oral or intravenous topotecan, a topoisomerase inhibitor, as second-line therapy besides treatment regimens with cyclophosphamide, doxorubicin, and vincristine [223, 224]. The antibody–drug conjugate Rovalpituzumab tesirine (Rova-T) targeting DLL3 proved to be less efficient than initially aspired, and further investigations on its efficacy based on molecular subtype stratification are warranted [225, 226]. Another noteworthy drug for second-line treatment of metastatic SCLC patients which has shown tolerability in initial trials is the selective RNA polymerase II inhibitor lurbinectedin [227]. Lurbinectedin has achieved accelerated approval by the FDA for SCLC patients with progression after first-line platinum-based chemotherapy [228].

Notably, several experimental therapeutic concepts have been recently introduced. SLFN11 expression has been recently demonstrated to predict response to lurbinectedin [229]. Indeed, multiple human SCLC cell lines sensitive to this drug have been described to express high levels of SLFN11. This finding was confirmed using siRNA knockdown of SLFN11 in mouse xenograft models [230]. The alkylating agent inducing double-strand breaks can be administered as a monotherapy or in combination with the ataxia telangiectasia and Rad3-related protein (ATR) inhibitors ceralasertib and berzosertib. Concerning the therapeutic efficacy, the synergistic effect of combination therapy has been shown to correlate with altered cell cycle regulation, resulting in mitotic catastrophe and apoptosis [229, 230]. Accordingly, the efficacy of combination therapy with lurbinectedin and berzosertib is currently being investigated in SCLC patients in a phase I/II clinical trial (NCT04802174). Similarly, the combination of berzosertib and the topoisomerase I (TOP1) inhibitor irinotecan exerts synergistic potential in SCLC due to high replication stress and yields objective response rates of 36% [231]. Of note, TOP1 and ATR interaction also modulates the activation of the stimulator of interferon genes (STING) pathway [232]. SCLC exhibits less intrinsic expression of STING than the normal lung epithelium and differential STING expression patterns are mainly associated with altered immune infiltration, EMT, or DNA damage response. The latter has been correlated with a STING-low SCLC phenotype, resulting in increased expression of pro‐inflammatory chemokines and cytokines as well as genes encoding interferon type I signaling following dual inhibition of TOP1 and ATR [232]. Likewise, lurbinectedin was shown to induce PD-L1 expression via activation of the cyclic GMP-AMP synthase (cGAS)-STING DNA sensing pathway, indicating a coherence with immunomodulation [229]. The recent proteomic analysis of 112 treatment-naïve SCLCs revealed that mutations in the zinc finger homeobox 3 (ZFHX3) gene encoding a transcription factor that transactivates the cell cycle inhibitor CDKN1A increases immune infiltration and contributes to immune-hot SCLCs [164]. Likewise, proteins associated with the cGAS-STING pathway were also elevated in immune-hot SCLCs. In contrast, immune-cold tumors displayed a positive correlation to DDR pathways and cytoplasmic nucleic acid sensing pathways which are crucial in tumors characterized by exceptionally high genomic instability [233]. Notably, these findings emphasize a potential immunosuppressive role of enhanced DDR activity by inhibiting the cGAS-STING pathway which is associated with immune infiltration and triggered by cytoplasmic double-strand DNA [164]. A divergent approach recently unveiled increased sensitivity to cisplatin and anti-PD-L1 therapy due to high expression of the pyroptosis-related protein gasdermin E (GSDME) and resulting release of IL12 cytokine and upregulation of IFN-γ in T-cells [234]. This interactive network promotes the transition from SCLC-cold into SCLC-hot tumors, thereby improving immunotherapeutic efficacy [234].

While targeted therapy is currently not part of SCLC´s therapeutic armamentarium, efforts are being made to identify subtype-specific targets. BET family proteins are key modulators of transcription and are being evaluated both in the preclinical and clinical settings. BET inhibitors (BETis) prevent the interaction of BET proteins and active chromatin, resulting in transcriptional regression. Since BET proteins have been associated with NEUROD1 expression, BET inhibition may display a subtype-specific therapy for SCLC-N. Recent preclinical data confirmed the direct interaction of NEUROD1 and BET proteins including BRD4 (Bromodomain-containing protein 4) and MED1 (Mediator Complex Subunit 1) [235]. Accordingly, SCLC-N cell lines are the most susceptible to the BETis JQ1 and OTX-015. The potent BETi NHWD-870 was used to obtain in vivo validation due to its superior pharmacokinetics compared to JQ1, and it is currently being investigated in early-phase clinical trials. In SCLC-N lines, treatment with NHWD-870 resulted in decreased tumor burden. However, resistance to BETis frequently occurred [235]. Moreover, combinational approaches including BET and mTOR inhibitors are being tested for potentiation of anti-tumor efficacy in SCLC. Dual targeting of SCLC PDX models increased anti-tumor activity without severe toxicity [236]. The drug interaction has been narrowed down to increased RSK3 expression which increases survival via stimulation of the TSC2-mTOR-p70S6K1-BAD cascade [236]. A phase I/II clinical trial is currently recruiting SCLC patients for the treatment with sirolimus plus auranofin (targeting mTOR and thioredoxin (TRX) reductase). Prior to this, NE SCLC cell lines showed selective vulnerability to TRX antioxidant pathway inhibition. Consequently, subtype-specific responses may obscure potential positive clinical trial outcomes, highlighting the need for molecular characterization and enabling appropriate patient selection [104].

Since ASCL1 and NEUROD1 expression occurs in ≈ 86% of SCLC cases and both TFs are yet not directly targetable, the focus is laid on indirect personalized approaches. The nuclear shuttle of both NE TFs is mediated by karyopherin β1 (KPNB1) [237]. The selective nuclear import of NEUROD1 is accompanied by the shuttle of its E-Box binding partner TCF3. Additionally, the nuclear translocation of ASCL1 via KPNB1 is facilitated after heterodimerization with TCF3 [237]. Consequently, nuclear deprivation of ASCL1 and NEUROD1 via KPNB1 inhibition led to impaired growth of SCLC-A and SCLC-N tumor cells in vitro and tumor shrinkage of ASCL1-driven xenografts in vivo [237]. In summary, the clinical importance of nuclear transporters for addressing NE SCLC subtypes needs further investigation.

Anti-apoptotic proteins of the BCL2 family are frequently dysregulated in SCLC specimens [163]. There is no molecular subtype that is mutually exclusive, though both SCLC-A and SCLC-P show elevated expression levels of BCL2 [238]. Although high BCL2 levels partly predict sensitivity to the BCL2 inhibitor venetoclax, not all SCLC cell lines respond accordingly. Decreased BCL2-associated X protein (BAX) expression and concomitant overexpression of MCL1 were characteristic for venetoclax-resistant cell lines. Hence, the combination of venetoclax and S63845 (MCL1 inhibitor) resulted in synergistic drug interactions in vitro and in vivo in double-resistant SCLC cell lines, but only when BAX expression was detectable. Induction of ectopic BAX in non-responding cell lines ultimately sensitized cells to combined venetoclax and S63845 therapy [238]. Similarly, co-targeting of BCL-X_L_ and MCL1 in SCLC cell lines using DT2216 (BCL-X_L_ degrader) and AZD8055 (mTOR inhibitor) showed synergistic responses [239]. Based on downregulation of MCL1, the dual targeting induced apoptosis and tumor regression in SCLC xenograft models. Moreover, superior survival and decreased tumor burden have been validated in GEMMs of SCLC [239]. Altogether, the genomic and proteomic profiles of SCLC including anti-apoptotic proteins govern drug response and resistance mechanisms, but hold the potential of novel personalized therapy.

## Conclusions

SCLC remains a highly recalcitrant and lethal cancer characterized by distinct molecular patterns and concomitant high tumoral heterogeneity. Compared to other malignancies, preclinical SCLC research and thus translational success have been significantly hampered by the lack of representative patient material. Nevertheless, great advancements in characterizing the origin and the genetic and molecular features of SCLC have been made in the past years. In 2019, Rudin et al. disclosed non-NE subtypes besides the renowned NE phenotypes characterized by ASCL1 and/or NEUROD1 expression, leading to further investigation and the definition of another, “inflamed” subtype. These molecular subclassifications revealed several underlying characteristics and variances in SCLC, thereby opening up numerous novel avenues to be preclinically explored. There is great hope that the implementation of preclinical in vitro and in vivo state-of-the-art methodology and tools, such as organoids and genetically engineered/humanized mouse models, will continue to provide valuable assets in uncovering therapeutically targetable vulnerabilities that can be implemented in clinical practice in the next years. In the clinics, however, SCLC is still considered a homogenous disease. Therefore, patients are still being treated in an untargeted manner irrespective of their tumor's molecular characteristics. Thus, considering the broad range of preclinical approaches for personalized medicine in SCLC, patient stratification will be of necessity to evaluate proper anti-tumor efficacies. To this end, adequate experimental platforms to improve material collection and collaboration efforts to share valuable material and data, together with clinical trials evaluating novel treatment strategies in suitable patients are urgently warranted in order to improve SCLC patient outcomes.

## Data Availability

Not applicable.

## Abbreviations

AJUBA: Ajuba LIM protein
ALDH: Aldehyde dehydrogenase
ALK: Anaplastic lymphoma kinase
AMPK: AMP-activated protein kinase
ANNA: Anti-neuronal nuclear antibody
APC: Antigen-presenting cells
ASCL1: Achaete-scute complex homolog 1
AT1: Alveolar type 1 cells
AT2: Alveolar type 2 cells
ATR: Ataxia telangiectasia and Rad3-related protein
AURK: Aurora kinase
BADJ: Bronchioalveolar duct junction
BASC: Bronchioalveolar stem cell
BAX: BCL2-associated X protein
BER: Base excision repair
BCL2: B-cell lymphoma 2
bHLH: Basic-helix-loop-helix
BRD4: Bromodomain-containing protein 4
CALCA: Calcitonin Related Polypeptide Alpha
CAR-T: Chimeric antigen T cell transfer
CDH1: E-cadherin
CDKN1A/p21: Cyclin dependent kinase inhibitor 1A
CDX: Cell-derived xenografts
cGAS: Cyclic GMP-AMP synthase
CGRP: Calcitonin gene-related peptide
CHGA: Chromogranin A
CHT: Chemotherapy
CREBBP: CREB-binding protein
CSC: Cancer stem cell
ctCDX: Circulating tumor cell-derived xenograft
ctDNA: Circulating tumor DNA
CTLA4: Cytotoxic T-lymphocyte-associated protein 4
DHH: Desert Hedgehog
DLL3: Delta like canonical notch ligand 3
DNMT: DNA methyltransferase
Dpf: Days post fertilization
EGFR: Epidermal growth factor receptor
EMT: Epithelial-to-mesenchymal transition
EpCAM: Epithelial cell adhesion molecule
EZH2: Enhancer of zeste homologue 2
FGF(R): Fibroblast growth factor (receptor)
GD2: Glycolipid tumor antigen 2
GEMM: Genetically engineered mouse model
GFI1B: Growth factor independence 1B
GLI1/2/3: Glioma-associated oncogene 1/2/3
GRP: Gastrin-releasing peptide
GSDME: Gasdermin E
GSH: Glutathione
HAT: Histone acetyltransferase
HDAC: Histone deacetylase
HES1: Hairy and enhancer of split-1
HEY1: Hairy and enhancer of split-related protein 1
HH: Hedgehog
HLAs: Human leukocyte antigens
ICI: Immune checkpoint inhibition
ID2: Inhibitor of Differentiation 2
IFN-γ: Interferon-γ
IGF1R: Insulin-growth factor receptor 1
IHH: Indian Hedgehog
IL12: Interleukin 12
INSM1: Insulinoma-associated 1
JLL1/2: Jagged-like ligands 1/2
KDM6A: Lysine Demethylase 6A
KPNB1: Karyopherin β1
LATS1/2: Large tumor suppressor kinase 1/2
LSD1: Lysine demethylase 1A
LUAD: Lung adenocarcinoma
MED1: Mediator complex Subunit 1
MET: Mesenchymal-epithelial transition
MDR: Multidrug resistance
MLL1/4: Histone methyltransferases
MOB1: MOB kinase activator 1
MST1/2: Mammalian sterile 20-like kinases 1/2
mTOR: Mammalian Target of Rapamycin
MVA-GGPP: Mevalonate-geranyl-geranyl-diphosphate
NCAM1: Neural cell adhesion molecule 1
NE: Neuroendocrine
NEBs: Neuroepithelial bodies
NER: Nucleotide excision repair
NEUROD1: Neurogenic differentiation factor 1
NFIB: Nuclear factor 1B
NGS: Next-generation sequencing
NICD: Notch intracellular domain
NSE: Neuron-specific enolase
OS: Overall survival
PARP: Poly(ADP-ribose) polymerase
PD-L1: Programmed death-ligand 1
PDX: Patient-derived xenografts
PFS: Progression-free survival
PNECs: Pulmonary neuroendocrine cells
POU2AF2: POU Class 2 Homeobox Associating Factor 2
POU2AF3: POU Class 2 Homeobox Associating Factor 3
POU2F3: POU class 2 homeobox 3
PROX1: Prospero homeobox protein 1
PTCH1/2: Patched 1/2
PTEN: Phosphatase and tensin homolog
RARB: Retinoic acid receptor beta
RB1: Retinoblastoma 1
RBL1: Retinoblastoma-Like Protein 1
REST: RE1 silencing transcription co-repressor
RRA: Rapid research autopsy
SAV1: Salvador homolog 1
SCLC (A/N/P/Y/I/QN): Small cell lung cancer (ASCL1, NEUROD1, POU2F3, YAP1, inflamed, quadruple negative)
SHH: Sonic hedgehog
SLFN11: Schlafen family of proteins
SMO: Smoothened
SOX2: SRY-box transcription factor 2
STING: Stimulator of interferon genes
SUFU: Suppressor of fused
SYP: Synaptophysin
TAZ: Transcriptional Coactivator with PDZ-binding Motif
TF: Transcription factor
TGF-β(R): Transforming growth factor-β (receptor)
TKI: Tyrosine kinase inhibition
TKO: Triple knock-out
TMB: Tumor mutational burden
TME: Tumor microenvironment
TOP1: Topoisomerase 1
TP53: Tumor protein 53
TRKB: Tropomyosin-related Kinase B
TRX: Thioredoxin
TS: Tumor suppressor
TTF1: Homeobox protein Nkx2.1
XPO1: Exportin-1
YAP1: Yes-associated protein 1
